# H3K4me3 inversely correlates with DNA methylation at a large class of non-CpG-island-containing start sites

**DOI:** 10.1186/gm346

**Published:** 2012-05-28

**Authors:** Dheepa Balasubramanian, Batool Akhtar-Zaidi, Lingyun Song, Cynthia F Bartels, Martina Veigl, Lydia Beard, Lois Myeroff, Kishore Guda, James Lutterbaugh, Joseph Willis, Gregory E Crawford, Sanford D Markowitz, Peter C Scacheri

**Affiliations:** 1 Department of Genetics and Genome Sciences, Case Western Reserve University, 10900 Euclid Ave, Cleveland, OH 44106, USA; 2 Department of Molecular Medicine, Cleveland Clinic Lerner College of Medicine, Case Western Reserve University, 9500 Euclid Ave, Cleveland, OH 44195, USA; 3 Institute for Science and Policy, and Department of Pediatrics, Duke University, 101 Science Drive, Durham, NC 27708, USA; 4 Case Comprehensive Cancer Center, Case Western Reserve University, 11100 Euclid Ave, Cleveland, OH 44106, USA; 5 Department of Pathology, Case Western Reserve University, 2103 Cornell Road, Cleveland, OH 44106, USA; 6 Department of Medicine, Case Western Reserve University, 10900 Euclid Ave Cleveland, OH 44106, USA

## Abstract

**Background:**

In addition to mutations, epigenetic silencing of genes has been recognized as a fundamental mechanism that promotes human carcinogenesis. To date, characterization of epigenetic gene silencing has largely focused on genes in which silencing is mediated by hypermethylation of promoter-associated CpG islands, associated with loss of the H3K4me3 chromatin mark. Far less is known about promoters lacking CpG-islands or genes that are repressed by alternative mechanisms.

**Methods:**

We performed integrative ChIP-chip, DNase-seq, and global gene expression analyses in colon cancer cells and normal colon mucosa to characterize chromatin features of both CpG-rich and CpG-poor promoters of genes that undergo silencing in colon cancer.

**Results:**

Epigenetically repressed genes in colon cancer separate into two classes based on retention or loss of H3K4me3 at transcription start sites. Quantitatively, of transcriptionally repressed genes that lose H3K4me3 in colon cancer (K4-dependent genes), a large fraction actually lacks CpG islands. Nonetheless, similar to CpG-island containing genes, cytosines located near the start sites of K4-dependent genes become DNA hypermethylated, and repressed K4-dependent genes can be reactivated with 5-azacytidine. Moreover, we also show that when the H3K4me3 mark is retained, silencing of CpG island-associated genes can proceed through an alternative mechanism in which repressive chromatin marks are recruited.

**Conclusions:**

H3K4me3 equally protects from DNA methylation at both CpG-island and non-CpG island start sites in colon cancer. Moreover, the results suggest that CpG-rich genes repressed by loss of H3K4me3 and DNA methylation represent special instances of a more general epigenetic mechanism of gene silencing, one in which gene silencing is mediated by loss of H3K4me3 and methylation of non-CpG island promoter-associated cytosines.

## Background

The development of cancer is closely associated with the stepwise accumulation of not only somatic mutations, but also epigenetic alterations that alter chromatin structure and lead to dysregulated gene expression. Current dogma holds that for normal somatic cells, trimethylated lysine 4 on histone H3 (H3K4me3) represents a chromatin landmark that is present at the transcription start sites (TSSs) of protein-coding genes that are either actively transcribed or that are held in a 'poised' state permissive for gene transcription [1]. However, it is also well established that during the process of malignant transformation, loss of H3K4me3 occurs at TSSs of genes that undergo transcriptional inactivation as a result of promoter hypermethylation [2,3]. The loss of H3K4me3 is consistent with a model whereby DNA methylation at CpG islands is initiated by removal of H3K4me by LSD1 (lysine-specific demethylase 1) and JmjC families of demethylases, followed by targeting of DNA methyltransferase (DNMT)3A/Dnmt3B-Dnmt3L complexes, which deposit methyl groups [2,4-7]. However, *de novo *DNA methylation is an infrequent event occurring on only a very small fraction of CpG island-containing promoters. Far less is known about promoters lacking CpG islands or genes that are repressed by alternative mechanisms, mainly because genome-wide surveys of epigenetic modifications have only recently become technically feasible.

Here, we used ChIP-chip and expression analyses to systematically study the relationship between H3K4me3 and genes that are transcriptionally repressed in colon cancer compared to normal colon mucosa. We found that the majority of protein-coding genes contain H3K4me3, as expected. Interestingly, H3K4me3 is retained among a set of genes that undergo transcriptional repression, or 'silencing' during the process of malignant transformation. Repressed genes that retain H3K4me3 are also located in open regions of chromatin that are hypersensitive to DNaseI digestion, nearly always contain CpG islands, and frequently acquire histone modifications associated with transcriptional repression. Consistent with the established inverse correlation between DNA methylation and H3K4me3, we also detected a class of repressed genes that virtually lack detectable levels of H3K4me3 and show increased DNA methylation compared to normal colon mucosa. While the increased DNA methylation accompanying loss of H3K4me3 is easily detected at promoters containing CpG islands, we find this increase is often more prevalent at the scattered CpG sites in the promoters of genes devoid of CpG islands. We propose a model whereby H3K4me3 equally protects from DNA methylation at both CpG island and non-CpG island start sites, suggesting that the mechanisms associated with DNA methylation-associated gene silencing in colon cancer are similar for CpG and non-CpG island-containing genes.

## Materials and methods

### Cell lines and tissue samples

The VACO cell lines (VACO429, VACO432, VACO441 and VACO425) were cultured as previously described [8]. SW480 was obtained from the American Type Culture Collection. Normal colon mucosa was obtained from a scraping of fresh resected colon. Cell viability was examined by staining with Trypan Blue, and estimated at 85 to 95%. Extraction of colonic crypts was performed by EDTA fractionation. Briefly, colon mucosa was first dissected from each sample taking care to maintain tissue integrity. The mucosa was then cut into thin strips, gently agitated in cell dissociation buffer (Invitrogen 13151-014 Carlsbad, CA, USA), rinsed in phosphate-buffered saline, and then agitated in fresh buffer. The tissue strips were then rapidly pipetted until the solution became cloudy due to the release of crypts from the mucosa. Mucosa strips were removed from the solution and discarded. The remaining solution was examined microscopically for the presence of viable, intact crypts free of contaminating debris.

### ChIP-chip

Chromatin immunoprecipitation (ChIP)-chip experiments were performed as previously described [9] using the following antibodies: H3K4 trimethyl (Abcam ab8580 (Cambridge, MA, USA); Upstate 39159 (Billerica, MA, USA)), H3K9 dimethyl (Abcam, ab1220), H3K20 trimethyl (Abcam ab9053), and H3K27 trimethyl (Abcam ab6002). The following three array platforms were used: (1) NimbleGen (Madison, WI, USA) human 2.1M deluxe promoter arrays; (2) NimbleGen 385K promoter arrays; and (3) Agilent (Santa Clara, CA, USA) custom tiling arrays. The NimbleGen human 2.1M promoter array contains 50- to 70-mer probes spanning 7.2 kb upstream and 3.2kb downstream of each TSS at a resolution of 1 oligo per 100 bp. NimbleGen 385K arrays contain 50- to 70-mer probes spanning 1 kb upstream and 500 bp downstream of approximately 19,000 TSSs at a resolution of 1 oligo per 100 bp. To account for potential differences in the dyes on the array, ChIP/input ratios for each probe were scaled by subtracting the bi-weight mean for the log-ratio values from each log-ratio. ChIP-chip signals were then quantile normalized so that separate ChIP-chip experiments could be directly compared. Agilent microarrays contained 44,000 features, with oligos that spanned 10 kb downstream and 5 kb upstream of the TSS of the desired genes. Agilent array data were processed and normalized using Feature Extraction. For the comparison of multiple histone marks between normal colon mucosa and SW480, ChIP-chip data were processed with the software package ACME using a window size of 500 bp and a threshold of 95% [9]. The minimum *P*-values for signals located near (±1 kb from each TSS) were then calculated and plotted.

### Expression analyses

RNA was purified by cesium chloride density gradient centrifugation from all cell lines, normal colon mucosa, and normal colon crypt preparations. RNA was then labeled and hybridized to Affymetrix (Santa Clara, CA, USA) Human Exon 1.0 ST exon arrays according to standard protocols. All microarray data were processed in a single batch using the Affymetrix Expression Console software, obtaining gene level expression data using 'core' probe sets (the highest confidence level probe sets, associated with BLAT alignments of mRNA with annotated full-length coding sequence regions) using median normalization and the PLIER (probe logarithmic intensity error estimation) algorithm. Genes located on Y, random, and the mitochondrial chromosomes were excluded. Repressed genes were considered those with an expression value of <31, while genes with expression values >75 were considered expressed or 'on'. These thresholds were determined through analysis of the distribution of expression of all genes sampled on the microarray. K4-independent genes were designated as those repressed (<31) in the colon cancer cell lines compared to normal colon crypt (>75), and containing enrichment of H3K4me3 in normal colon mucosa and colon cancer cell lines at levels corresponding to the right side of the bimodal distribution of H3K4me3 levels of all genes. K4-dependent genes were designated as repressed genes (<31) that showed levels of H3K4me3 corresponding to the left side of the bimodal distribution.

### DNase-seq

DNase-seq was performed as previously described [10]. Sequencing was performed using an Illumina (San Diego, CA, USA) GAII, and 35-bp reads were obtained for all samples. Sequences were aligned to the human genome reference sequence (Hg18) using MAQ [11]. The number of aligned sequence reads were as follows: SW480, 21,461,814; V432, 16,003,416; V429, 7,110,678. Aligned sequences were processed with F-seq [12]. The maximum peak signal within 2 kb of all human TSSs was then extracted from the genome-wide DNase-seq profiles, so that each TSS could be assigned a single score. Scores were Z-score transformed so that individual samples could be directly compared.

### Methylation analysis

Pyrosequence analysis of bisulfite converted and non-converted DNA was performed by EpigenDx (Worcester, MA, USA). CpG sites analyzed were located at the following positions relative to the TSSs of each indicated gene (HG19 genome assembly): *MMP28*, (-589, -582, -563, -536, -504,-486); *PTGDR*, (55, 58, 68, 86, 124, 126, 130, 148, 150, 153, 162, 166,175); *HMGCS2*, (59, 78, 98, 125); *ACSL5*, (22, 28, 55, 97, 117, 150, 154, 176, 200); *BCAS1*, (-262, -223, -195, -132); *FRK*, (-503, -493, -484, -442, -431); *UBA7*, (-123, -134); *BCL2L14*, (55, 19); *PIGR*, (-673, -656, -650, -597); *CD177*, (-150, -6, 5, 7); *GUCY2C*, (-124, -161, -126); *TNFSF10*, (-43, -98); *SEMA6D*, (-94, -90, -81, -76, -60, -57, -50, -42); *MOBKL2B*, (-77, -71, -67, -54, -50, -47, -41, -39, -30, -23, -19, -13, -11, -8, -6); *SLC39A5*, (-297, -218, -162, -102, -50).

### 5-Azacytidine treatment

SW480 cells were seeded at 10^5 ^per T75 flask on day 0. The cultures were treated for 24 h on days 2 and 5 with 5-azacytidine at 1 µg/µl. The media was changed 24 h after the addition on 5-azacytidine (on days 3 and 6). RNA harvested 8 days after the initial 5-azacytidine treatment was DNase-treated and purified using the RNeasy kit (Qiagen, Germantown, MD, USA). cDNA was prepared using the high capacity cDNA archive kit (Applied Biosystems, Foster City, CA, USA), and standard quantitative RT-PCR analyses were performed.

## Results

### H3K4me3 ChIP-chip in colon cancer cell lines

**Figure 1:**
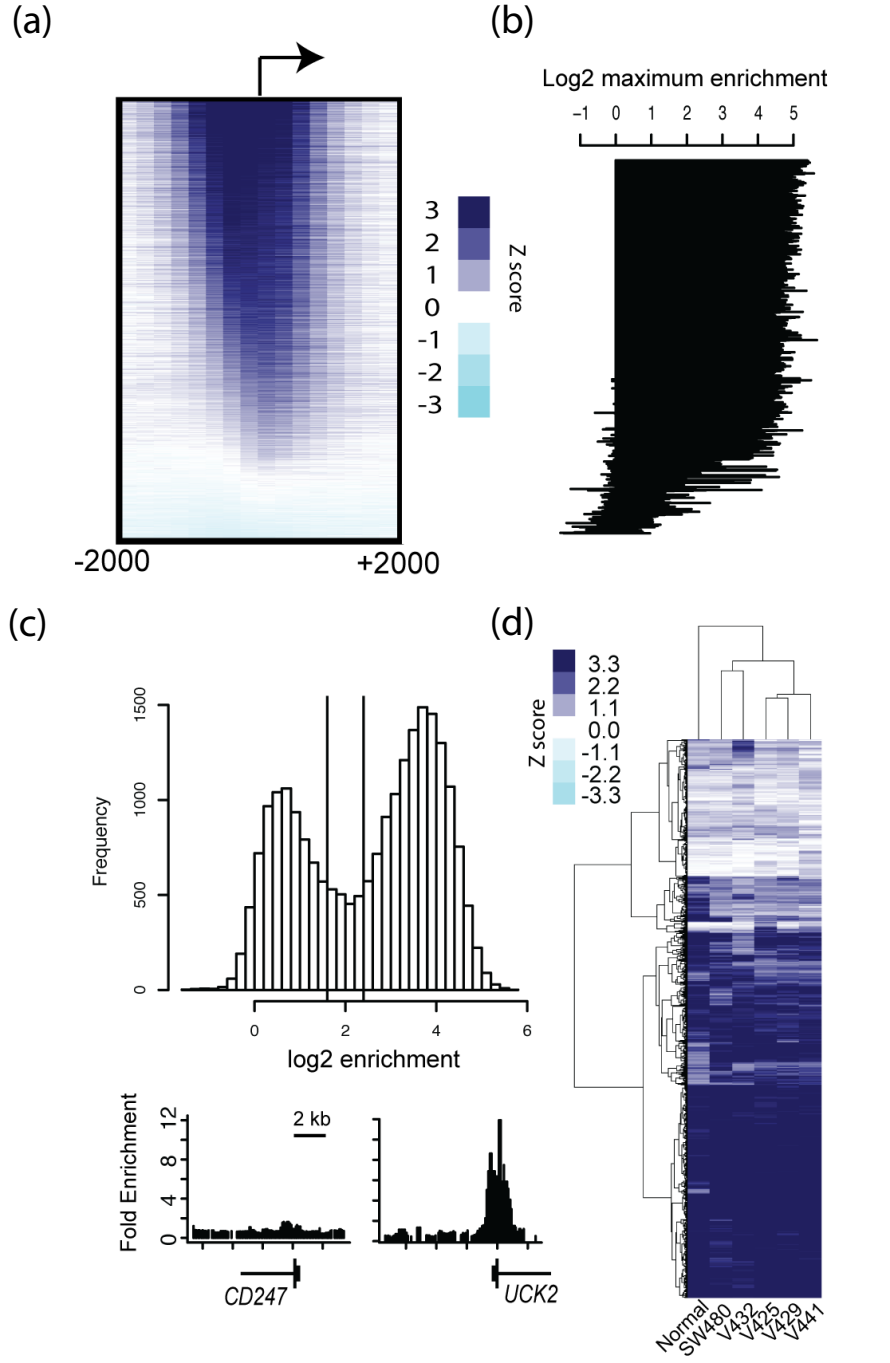
**ChIP-chip of H3K4me3**. **(a) **Enrichment of H3K4me3 within 2 kb of all human TSSs. **(b) **Maximum enrichment of H3K4me3 within 1 Kb of the TSS for each human gene. Genes are ordered similarly to (a). **(c) **Histogram of H3K4me3 signals at all human TSSs. The vertical lines correspond to thresholds used to distinguish genes containing high levels of H3K4me3 (right-side of the distribution) from genes containing low H3K4me3 levels (left side of the distribution). Genes falling between the two vertical lines are indeterminate and not designated as K4-absent or K4-present genes. The bottom panel shows an example of a gene lacking H3K4me3 (*CD247*), and a gene containing H3K4me3 (*UCK2*). **(d) **Maximum H3K4me3 signal at all TSSs in the five colorectal cell lines (SW480, V432, V425, V429, V441) and normal colon mucosa. Columns represent individual samples, and rows represent H3K4me3 signals at TSSs. Dark blue corresponds to high H3K4me3 enrichment; light blue corresponds to little or no enrichment.

We performed ChIP studies using microarrays containing 2.1 million oligonucleotides tiled across all human promoters to define the repertoire of genes containing H3K4me3. These studies were carried out in five colon cancer cell lines (SW480, V432, V425, V429, V441) and normal colon mucosa. A representative view of the H3K4me3 ChIP-chip data for one of the six samples tested is shown in Additional file 1. Similar to previous studies in human embryonic stem cells, primary hepatocytes and B cells [1], we found that the majority (57 to 66%) of all annotated promoters in colon were enriched for H3K4me3 at medium to high confidence (Figure 1a,b). Next we plotted the levels of H3K4me3 for all genes as a histogram. The data show a bimodal distribution with peaks at genes that have robust levels (right side of distribution) and weak or absent levels (left side of distribution) of H3K4me3 (Figure 1c). Examples of genes with H3K4me3 enrichment values at either peak of the bimodal distribution are shown in Figure 1c. Lastly, we performed a cluster analysis of the H3K4me3 promoter signals across all six samples. The results indicate that while the majority of promoters show similar H3K4me3 levels among different individual samples, a small fraction of promoter-specific differences between individuals are clearly apparent (Figure 1d).

### Repressed genes can be distinguished into two classes, based on the presence or absence of H3K4me3

We next set out to identify genes that are repressed in each of the colon cancer samples compared to normal colon mucosa. For these experiments, we prepared RNA from each of the five colon cancer cell lines, as well as microdissected histologically normal colon mucosa, and five individual preparations of epithelial crypts purified by fractionation from normal colon mucosa. Samples were hybridized to Affymetrix Human Exon 1.0 ST Arrays, which are known to be more reliable for transcript quantification than standard 3'-UTR microarrays. Similarly to the H3K4me3 data, the genome-wide distribution of gene expression is largely bimodal (Additional file 2A). This allowed us to divide genes into two main categories: (1) abundantly expressed or 'on'; and (2) near background levels or 'off'. Among all samples analyzed, we found that, on average, 49% of genes were expressed and 33% of genes were off; 18% of genes fell in the trough of the bimodal distribution and could not be neatly classified as either silent or expressed.

**Figure 2:**
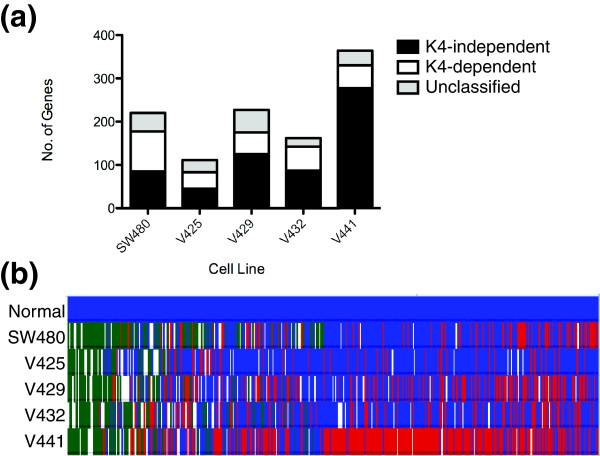
**Designation of K4-dependent and K4-independent genes in colon cancer**. **(a) **Bar plot showing proportions of K4-dependent and K4-independent genes in each of the five colon cancer cell lines. **(b) **Heatmap integrating H3K4me3 ChIP-chip signals and corresponding transcript levels. Blue, expressed and H3K4me3 present; red, not expressed and H3K4me3 present; green, not expressed and H3K4me3 absent; white, H3K4me3 status not classifiable.

Having identified the list of expressed and repressed genes, the next step was to merge the expression data to the ChIP-chip data. Because expression and ChIP-chip analyses were performed using different platforms, only genes represented on both platforms could be utilized for the combined analysis. Nevertheless, we identified >15,000 unique genes for which we had obtained both expression and H3K4me3 ChIP-chip data. We observed a high correlation between the expression and H3K4me3 levels in each of the cell lines (Additional file 2B). Using the combined datasets, we looked for genes that showed high expression and high levels of H3K4me3 in the normal colon samples, and were repressed in any one of the five colon cancer cell lines; 1,085 genes fit these criteria. We then examined the levels H3K4me3 among the repressed genes in each line. Surprisingly, a large portion of repressed genes (41 to 76%) contained significant levels of H3K4me3, while a much smaller fraction (15 to 34%) showed nearly absent levels of H3K4me3. We designated repressed genes that retain H3K4me3 as K4-independent and repressed genes that lose H3K4me3 as K4-dependent. The total number of K4-dependent and -independent genes varied substantially between each cell line, although in all samples more K4-independent genes were detected than K4-dependent genes (Figure 2a). Additionally, while there were some examples of genes that were repressed among all five colon cancer cell lines, by and large the identity of genes repressed differed between lines (Figure 2b). Despite this, genes designated as either K4-dependent or -independent in a given line generally showed the same designation when again repressed in any of the remaining five lines. While generally true, some exceptions to this generalization could be identified, that is, some K4-dependent genes were classified as K4-independent in a different line, and vice versa. This variability likely reflects molecular heterogeneity among the colon samples.

### Verification of K4-dependent and K4-independent classes

The levels of H3K4me3 for genes in each class were validated by standard ChIP on biological replicate samples (Additional file 3A). The loss of the H3K4me3 signal at K4-dependent genes cannot be due to homozygous deletions, as these regions could be successfully amplified by genomic PCR. For further verification, we performed hybridizations of H3K4me3 ChIPs from samples V425, V432, V441, SW480 and two independent preparations of normal colon mucosa to NimbleGen 385K promoter arrays and repeated the data analysis. Consistent with the previous results, both K4-dependent and -independent genes were evident, and the relative proportions of each class were similar within and between each cell line to the proportions found using the 2.1M feature arrays (Additional file 4A). Next, we examined the exon-tiling array data to confirm that genes designated as repressed were in fact repressed across all exons, and that when expressed in the crypt were expressed across all exons, consistent with the canonical transcript from the locus. We found that, compared to genes in crypt that were designated as 'on', and that conformed to the canonical transcript and its associated promoter, the expression levels across all exons of genes designated as repressed were at or near background levels. The exon usage across a representative example of a repressed gene is shown in Additional file 4B. Lastly, we investigated whether K4-dependent and K4-independent genes repressed in cell culture were similarly under-expressed in primary tumors. Using global expression data, we first selected K4-dependent and K4-independent genes that were repressed by at least two-fold in all five colon cancer cell lines relative to five epithelial colon crypt samples. We then determined the percentage of these genes that were also repressed in 120 primary tumors relative to 16 normal mucosa samples. Of all genes, only 7.7% of genes repressed in the cell lines are also repressed in tumors relative to mucosa, whereas 76% of K4-dependent and K4-independent genes repressed in cancer cell lines validated as repressed in primary tumors (*P *< 2.2 × 10^-16 ^by exact binomial test). Collectively, these data strongly support the existence of the two classes of repressed genes in colon cancer, indicate that alternative promoter usage is unlikely to account for the difference in H3K4me3 status between the two classes, and suggest that most genes identified as repressed in the cell culture models are genuinely repressed in colon cancer.

### K4-dependent and -independent genes show differences in chromatin structure

**Figure 3:**
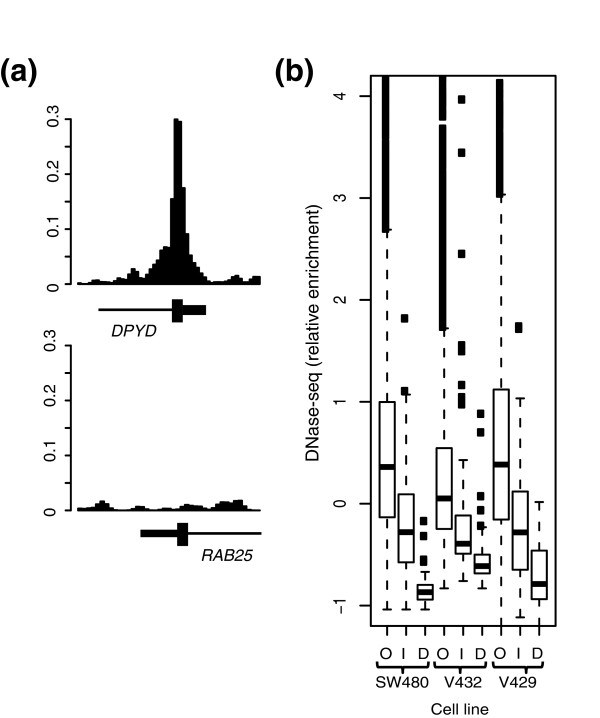
**Loss of H3K4me3 is associated with resistance to DNaseI digestion**. **(a) **Representative DNase-seq signals at a K4-independent (top) and K4-dependent (bottom) gene in cell line SW480. **(b) **Box plots depicting relative levels of DNase I hypersensitivity among expressed (O), K4-independent (I), and K4-dependent (D) genes.

We mapped open regions of chromatin in three of the five colon cancer lines (SW480 VACO432, and VACO429) using the technique of DNase-seq [10]. Each promoter was then assigned a score corresponding to the relative sensitivity to DNaseI digestion, and the data were merged to the H3K4me3 ChIP-chip data and expression data. We then tested whether promoters of K4-containing expressed genes, K4-dependent repressed genes, and K4-independent repressed genes differ in their sensitivity to DNaseI digestion. As expected, K4-containing expressed genes were significantly more sensitive to DNaseI digestion than both classes of repressed genes in all three cell lines (P < 5.7e-8; Figure 3). Of the two classes of repressed genes, K4-independent genes were significantly more sensitive to DNaseI digestion than K4-dependent genes (*P *< 0.0002). Specifically, 75 to 90% of genes designated as K4-independent were located within open chromatin, compared to 22 to 53% of K4-dependent promoters. Thus, compared to K4-independent genes, the promoters of repressed genes lacking H3K4me3 are generally located within relatively inaccessible conformations of chromatin.

### K4-dependent genes show DNA hypermethylation

**Figure 4:**
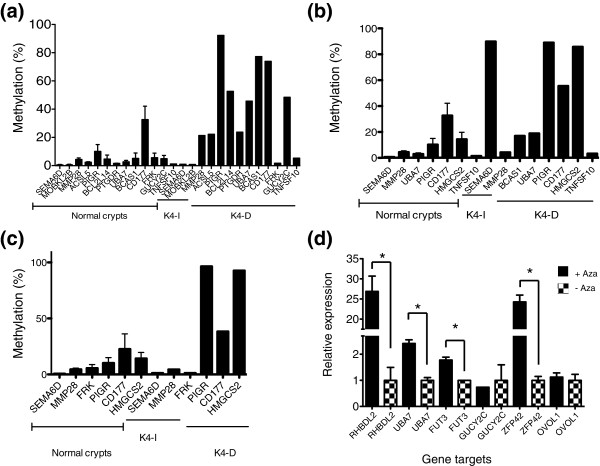
**K4-dependent promoters are DNA hypermethylated**. **(a-c) **Percentage DNA methylation of indicated K4-dependent (K4-D) and K4-independent (K4-I) genes in SW480 (a), V432 (b), and V441 (c). For comparison, methylation levels of genes were also quantified in normal colon crypts. Error bars indicate the standard deviation of pyrosequencing assays performed on three independent preparations of normal colon crypts. All K4-independent genes analyzed contain a CpG island at the TSS. All K4-dependent genes, except *PTGDR*, lack a CpG island at the TSS. **(d) **Quantitative RT-PCR analyses of genes from 5-azacytidine (Aza) treated (+) and untreated (-) SW480 cells. Data were normalized to *GAPDH*. *RHBDL2*, *UBA7*, *FUT3*, and *GUCY2C *are K4-dependent genes that lack a CpG island. *ZFP42 *was previously found in this cell line to be DNA hypermethylated and reversible upon treatment with 5-azacytidine, and thus serves as a positive control. *OVOL1 *is a K4-independent gene with a promoter-associated CpG island and serves as a negative control. Error bars indicate the standard deviation of quantitative RT-PCR reactions performed in triplicate. *P ≤ 0.01.

We next looked for the presence of CpG islands within 2 kb of the TSSs of K4-dependent and -independent genes; 95 to 99% of K4-independent genes were found to have a CpG island, compared to 33 to 56% of K4-dependent genes (Additional file 5). We noticed that several genes designated as K4-dependent in our study, including *CDX1*, *BMP3*, and *MLH1*, were previously reported to show CpG island promoter hypermethylation [13-15]. These findings prompted us to test whether K4-dependent genes lacking CpG islands also showed promoter hypermethylation. We performed pyrosequencing of bisulfite converted DNA to quantify DNA methylation at K4-dependent and K4-independent genes in cell lines in which these genes were repressed. As controls, these genes were also analyzed in three independent preparations of purified normal colon crypts. The pyrosequencing assays were designed to interrogate CpG sites located under the H3K4me3 peak of each gene, in close proximity (<700 bp) to the TSS. Nine of eleven K4-dependent repressed genes showed dramatic increases in DNA methylation over levels detected in normal colon crypt (Figure 4a-c). Moreover, several genes, including *PIGR*, *CD177*, and *HMGCS2*, were hypermethylated in more than one cell line in which these genes were designated as K4-dependent. In summary, 15 out of 19 assays performed on K4-dependent genes were positive for DNA hypermethylation. In comparison, two out of eight pyrosequencing assays performed on K4-independent genes were positive for DNA hypermethylation. These proportions are significantly different (*P *< 0.03 by Z-test for proportions).

We next tested whether K4-dependent genes that lack CpG islands could be reactivated upon treatment with 5-azacytidine. Three out of four K4-dependent genes tested showed a significant increase in transcript levels upon treatment with 5-azacytidine (*P *≤ 0.01; Figure 4d), consistent with hypermethylation of the scattered CpG sites under the H3K4me3 mark being functionally involved in these genes' silencing. The data indicate that DNA hypermethylated K4-dependent repressed genes do not necessarily contain CpG islands, and that repressed K4-dependent genes lacking both CpG islands and H3K4me3 are very likely to be DNA methylated in regions that lose the H3K4me3 mark. The results are also consistent with previously reported reactivation of hypermethylated genes lacking CpG islands upon treatment with 5-azacytidine [16].

### Characterization of histone marks at K4-independent and K4-dependent genes

**Figure 5:**
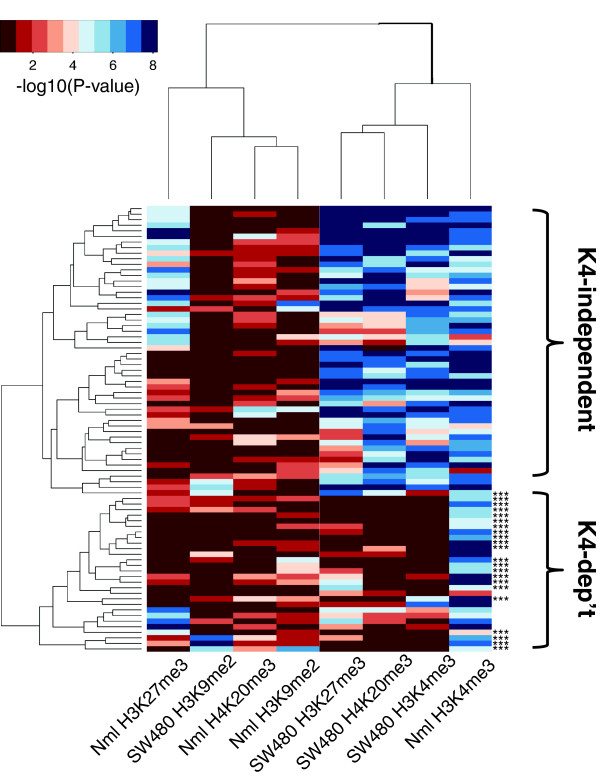
**ChIP-chip analysis of multiple histone marks at the promoters of repressed genes**. Plotted in the heatmap are -log10 *P*-values corresponding to ChIP-chip signal intensities of H3K4me3, H3K27me3, H3K9me2, and H4K20me3 at the promoters of 80 genes as measured in SW480 versus normal colon mucosa. The heatmap reveals two main classes of genes. Most repressed genes designated as K4-independent retain H3K4me3 (blue color) and gain repressive histone marks, including H3K27me3 and H4K20me3, which move from mostly red in normal to blue in the tumor. In contrast, most repressed genes designated as K4-dependent show loss of H3K4me3 (with H3K4me3 going from blue in tumor to red in SW480) and have not acquired any other repressive marks (with the other marks displayed as red in both the normal and tumor). In addition, the majority of genes within K4-dependent lack a CpG island (***no CpG island), while all K4-independent genes have a CpG island.

We performed ChIP-chip analysis to test whether repressed genes acquire histone modifications generally associated with transcriptional repression (H3K9me2, H3K27me3, and H4K20me3) and, if so, whether these modifications differ between K4-dependent and -independent genes. These studies were performed using custom-designed tiled arrays spanning the promoters and bodies of 80 genes repressed in colon cancer line SW480. ChIP-chip signal intensities for each mark and for each gene promoter were then hierarchically clustered and plotted in a heatmap. Strikingly, this analysis revealed a near perfect division of the K4-independent and K4-dependent gene classes (Figure 5). Moreover, consistent with data above indicating that K4-dependent genes often lack CpG islands, a minority (27%) of genes classified as K4-dependent were found to have a CpG island at the TSS, compared to nearly all (>95%) of the genes classified as K4-independent. In addition, we found that genes associated with repressive histone marks, including H3K27me3, H4K20me3, and occasionally H3K9me2, were mostly K4-independent genes in which the H3K4me3 mark had been retained, or were originally marked with both H3K4me3 and H3K27me3 in colon mucosa, so-called 'bivalent' chromatin [17]. The presence of H3K27me3 was validated at several K4-independent genes in SW480, as well as V441 and V429 by standard ChIP (Additional file 3B). Interestingly, with the exception of a few genes in our K4-dependent group that showed low-level acquisition of H3K27me3 or H3K9me2, the loss of H3K4me3 was usually not accompanied by any additional chromatin modifications (other than our finding of increased DNA methylation). While we currently cannot rule out the possibility that these K4-dependent genes acquired a repressive mark that was not tested, the data indicate that the repertoire of histone marks at K4-dependent and -independent genes are often distinct.

### K4-dependent and -independent genes are functionally distinct

We used Panther to determine whether specific pathways or biological processes are enriched among K4-dependent and -independent genes, and if so, whether they differ between the two classes [18]. Intriguingly, several pathways previously linked to colorectal carcinogenesis were enriched in the K4-independent set, including transforming growth factor-beta, insulin and Wnt signaling (Additional file 6). In contrast, K4-dependent genes were enriched in axon guidance, pyrimidine metabolism and cadherin signaling. Both classes were enriched for genes involved in apoptosis and platelet-derived growth factor signaling, as well as pathways associated with B- and T-cell activation.

We next tested whether genes in each class show tissue-specific expression in colon crypts, and if so, whether the degree of crypt-specific expression differed between the two classes. To do this, we compared global gene expression levels between normal colon crypt, HepG2, K562, and NB4 cells and computed a tissue-specificity score for each gene using the method of Shannon entropy [19]. We then plotted the distribution of colon-specificity scores for K4-dependent and -independent repressed genes, all genes, and 1,000 randomly selected genes. Both K4-dependent and -independent genes showed a high degree of crypt tissue-specific expression, with K4-dependent genes being significantly more crypt-specific than K4-independent genes (*P *< 0.0001) (Additional file 7A). These findings are consistent with other studies showing that genes lacking CpG islands, which comprise a large fraction of the K4-dependent class of repressed genes, are generally associated with tissue-specific expression [19].

Lastly, we tested whether K4-dependent genes show an increased propensity for silencing in colon cancer compared to K4-independent genes, or vice versa. To do this, the transcriptional status of genes designated as K4-dependent or K4-independent in the five colon cell lines was surveyed in an additional 35 colon cancer cell lines. We then plotted the distribution of the median expression values for each set as a histogram (Additional file 7B). Although the expression of K4-dependent genes is more variable than K4-independent genes, the overall distribution of K4-dependent genes is significantly shifted to the left of that of K4-independent genes (*P *= 0.015), indicating that K4-dependent genes are repressed more often in colon cancer than K4-independent genes. Although confirmatory studies are required, these findings raise the possibility that genes targeted for silencing in colon cancer are more often inactivated by mechanisms involving removal of H3K4me3 than by K4-independent mechanisms.

## Discussion

**Figure 6:**
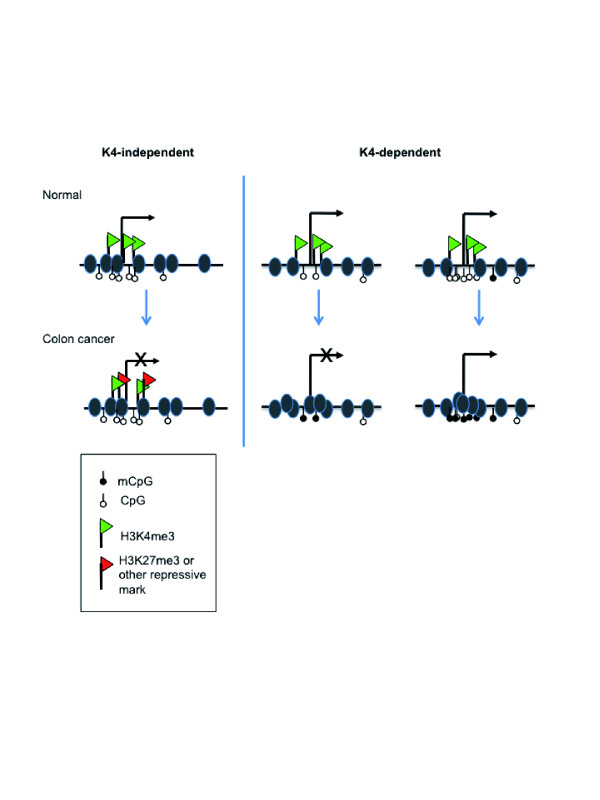
**Model of K4-dependent and K4-independent gene silencing in colon cancer**. Expressed genes in normal colon mucosa have high levels of H3K4me3, are DNA hypomethylated at the TSS, and are contained within open chromatin (grey ovals). K4-independent repressed genes retain appreciable levels of H3K4me3, often acquire repressive histone marks such as H3K27me3, and are often not DNA hypermethylated. Also, the chromatin at the TSS of K4-independent repressed genes is more condensed than in normal colon. Virtually all of these genes have a CpG island at the TSS. In comparison, K4-dependent repressed genes lack H3K4me3, are located in highly compacted chromatin, and are DNA hypermethylated, regardless of the presence of a CpG island. K4-dependent genes also do not acquire repressive chromatin marks.

In this paper we propose a revision of the commonly described model of epigenetic silencing of genes in cancer. Studies on epigenetic silencing of genes in cancer have generally focused on silencing by aberrant DNA methylation of CpG island containing promoters, accompanied by loss of the H3K4me3 mark. Here, we show that it is actually non-CpG island-containing genes that are often characteristically repressed by loss of the H3K4me3 mark, accompanied by aberrant methylation of scattered CpG residues near the TSS. Moreover, we find that repressed genes containing CpG islands are most commonly associated with repressive histone marks, such as H3K27me3, with these genes retaining H3K4me3. The two classes of repressed genes are further distinguishable by function and sensitivity to DNase digestion, with gene promoters losing H3K4me3 located within relatively condensed chromatin compared to promoters that retain H3K4me3. A model summarizing these findings is shown in Figure 6.

Two studies in which epigenetic silencing marks were profiled in prostate cancer cells (PC3) revealed that loci marked with H3K27me3 are devoid of DNA hypermethylation, raising the possibility that gene silencing by H3K27me3 occurs independently of promoter methylation [20,21]. These findings are consistent with our results, with one notable exception. The studies by Kondo *et al*. [20] indicate that promoters marked with H3K27me3 in the absence of DNA methylation are mostly devoid of CpG islands, while the data presented here indicate that this trend is far more prevalent for CpG island-containing promoters. In fact, only a small fraction of the genes we designated as K4-dependent, which are frequently devoid of promoter CpG islands, were found to contain significant levels of H3K27me3. The discrepancy between our results and the published studies is currently not clear, but could be related to differences in cancer type.

Previous studies have shown that low CpG promoters display no significant correlation between gene activity and the abundance of methylated cytosines [2]. The discrepancy between these findings and our results is likely due to methodological differences related to transcript quantification. Here, mRNA transcript levels were directly quantified using all exon microarrays. In contrast, in the previous studies promoter activity was 'presumed' based on occupancy of RNA polymerase II, which is now known to bind both active and 'paused' promoters that are weakly transcriptionally active [1,22,23]. Our findings are consistent with more recent studies showing significant positive correlations between DNA hypermethylation and low gene activity at CpG-poor promoters in multiple human tissues [24].

We find it particularly intriguing that K4-dependent repressed genes, which lack H3K4me3, are apparently devoid of repressive histone modifications, including H3K27me3, H3K9me2, and H4K20me3. While we cannot rule out the possibility that these genes acquire a currently unknown repressive mark, one possibility is that the loss of H3K4me3, together with methylation of residual CpGs, is sufficient for gene silencing in cancer. Further studies in which H3K4me3 is restored at these genes' promoters could help test this hypothesis.

## Conclusions

We conclude that the presence of the H3K4me3 mark at low CpG-content TSSs protects from DNA methylation and transcriptional repression in colon cancer. Quantitatively, of transcriptionally repressed genes that lose H3K4me3 and become DNA hypermethylated in colon cancer, more typically lack CpG islands than contain CpG islands.

Based on these findings, we propose that CpG-rich genes repressed by loss of H3K4me3 and DNA methylation represent rare examples of a more general epigenetic mechanism of gene repression, one in which silencing is mediated by loss of H3K4me3 and methylation of non-CpG island promoter-associated cytosines. Lastly, we note that the identification of gene silencing in cancer in association with CpG island methylation has led to the discovery of 5-azacytidine as a drug able to reactivate expression of such genes and to the use of 5-azacytidine for cancer treatment. It will similarly be of interest to seek to identify other pharmacologic agents that induce re-expression of repressed genes that lack CpG islands.

## Abbreviations

bp: base pair; ChIP-chip: chromatin immunoprecipitation combined with microarray technology; DNAse-seq: DNase I hypersensitive site sequencing; DNMT: DNA methyltransferase; H3K4me3: histone H3 trimethylated at lysine 4; H3K9me2: histone H3 dimethylated at lysine 9; H4K20me3: histone H4 trimethylated at lysine 20; TSS: transcription start site.

## Competing interests

The authors declare that they have no competing interests.

## Authors' contributions

PCS conceived and designed the experiments. DB performed all ChIP analyses and 5-azacytidine experiments. DB, LS, and GEC performed the DNase-seq experiments. JL, JW, LB, and SDM provided samples. MV performed the expression analyses. BA-Z performed validation experiments. KG and CFB provided technical assistance. DB, BA-Z, LM, and PCS analyzed the data. DB, B-AZ, SDM, and PCS wrote the paper. All authors have read and approved the manuscript for publication.

## Supplementary Material

Additional file 1 **Figure S1 - results of H3K4me3 ChIP-chip analysis at a region on human chromosome 1**. The asterisk denotes a signal that is enlarged on the right. Click here for file

Additional file 2 **Figure S2**. (A) **Bimodal distribution of expression levels of genes in SW480, as determined by microarray analysis**. Genes with expression values less than 31 (left of the red line) were considered 'off'. Genes with expression values greater than 75 were considered 'on' (right of the blue line). Similar distributions were observed for all remaining cell lines tested. **(B) **Scatter plot of expression correlated with the H3K4me3 levels in SW480. Vertical and horizontal lines denote the thresholds used for analysis. Similar plots were observed for all remaining cell lines tested. **(C) **Quantitative real time PCR validation of microarray data. Expression data for three K4-independent (top left) three K4-dependent genes (top right) common to colon cancer lines SW480 and V429 are plotted relative for both lines and three control crypts, relative to the averages of the control crypt samples for each respective gene. Bottom: same for two genes overexpressed in SW480 and V429 relative to normal crypt samples. Click here for file

Additional file 3 **Figure S3. (A) Quantitative H3K4me3 ChIP of K4-independent (red) and K4-dependent genes (green) in colon line SW480 (bottom) and normal colon mucosa (top)**. The names of the genes tested are shown at the bottom of each plot. NC corresponds to a non-target control region. PSMB4 serves as a positive control. **(B) **Quantitative H3K27me3 ChIP in SW480, V441, and normal colon mucosa. K4-independent and -dependent genes are shown in red and green, respectively. NT, non-target controls. Click here for file

Additional file 4 **Figure S4. (A) Verification of K4-dependent and K4-independent gene classes using independent ChIP reactions and NimbleGen 385K promoter arrays**. **(B) **Integrated Genome Browser view showing exon usage for a gene expressed in SW480 (top) and a gene designated as repressed in SW480 (bottom). Data for three of five colon crypt preparations are shown. The y-axis in each of the plots corresponds to the relative intensity of microarray probes spanning each exon. The red horizontal lines denote the 'on' threshold for expression and are matched for all samples. Other genes designated as repressed showed similar results. Click here for file

Additional file 5 **Figure S5. CpG content of K4-dependent and K4-independent repressed genes**. Percentages of K4-dependent and -independent promoters with CpG islands in each of the colorectal cell lines are shown. *P < 0.001. Click here for file

Additional file 6 **Table S1. Panther analysis of K4-independent (I) and K4-dependent (II) genes**. Click here for file

Additional file 7 **Figure S6. Tissue specificity and the frequency of silencing of K4-independent and -dependent genes in colorectal cancer**. **(a) **All genes were assigned a score based on their specificity of expression in normal colon, compared to non-colon cells. Low scores correspond to a high degree of tissue-specificity. Shown in the plot are the distribution of tissue-specificity scores for K4-dependent and K4-independent genes, a set of randomly chosen genes, and all genes. Arrows denote the median scores for each gene set. **(b) **Histogram of expression values for K4-dependent and K4-independent genes in a set of 35 colon cancer lines. Vertical bars in each plot correspond to the median. Click here for file
