# Supplementary material for: H3K4me3 inversely correlates with DNA methylation at a large class of non-CpG-island-containing start sites
Source: Genome Med. 2012 May 28;4(5):47. doi: 10.1186/gm346 (PMC3506913; doi:10.1186/gm346)
Supplement: Additional file 6 — Table S1. Panther analysis of K4-independent (I) and K4-dependent (II) genes. [file gm346-S6.DOCX]

| **Pathway** | **Pathway** | **P Value** | **Biological Process** | **P Value** |
| --- | --- | --- | --- | --- |
| **Class I** | Apoptosis signaling pathway | 1.63E-05 | Developmental processes | 9.34E-12 |
|  | TGF-beta signaling pathway | 1.27E-03 | Cell cycle control | 1.98E-10 |
|  | PDGF signaling pathway | 1.45E-03 | Signal transduction | 3.71E-09 |
|  | Angiotensin II-stimulated signaling through G proteins and beta-arrestin | 2.09E-03 | mRNA transcription regulation | 6.51E-09 |
|  | B cell activation | 3.44E-03 | Intracellular signaling cascade | 1.12E-08 |
|  | Insulin/IGF pathway-protein kinase B signaling cascade | 5.26E-03 | Protein modification | 2.30E-08 |
|  | Wnt signaling pathway | 6.19E-03 | mRNA transcription | 3.66E-08 |
|  | Alzheimer disease-presenilin pathway | 1.07E-02 | Cell proliferation and differentiation | 4.27E-08 |
|  | T cell activation | 1.13E-02 | Protein phosphorylation | 1.07E-07 |
|  | Dopamine receptor mediated signaling pathway | 1.38E-02 | Oncogenesis | 1.93E-06 |
|  |  |  |  |  |
|  | Axon guidance mediated by semaphorins | 3.50E-03 | Oncogenesis | 2.90E-04 |
| **Class II** | Pyrimidine Metabolism | 3.86E-03 | Lipid, fatty acid and steroid metabolism | 4.69E-03 |
|  | T cell activation | 6.20E-03 | Receptor mediated endocytosis | 6.20E-03 |
|  | Apoptosis signaling pathway | 1.09E-02 | Fatty acid metabolism | 7.82E-03 |
|  | B cell activation | 1.90E-02 | Cell structure and motility | 8.14E-03 |
|  | Cadherin signaling pathway | 2.46E-02 | Oncogene | 8.56E-03 |
|  | Salvage pyrimidine deoxyribonucleotides | 2.56E-02 | Acyl-CoA metabolism | 9.22E-03 |
|  | PDGF signaling pathway | 3.55E-02 | Endocytosis | 9.86E-03 |
|  | Phenylethylamine degradation | 6.28E-02 | Detoxification | 1.14E-02 |
|  | Nicotine degradation | 6.28E-02 | Fatty acid beta-oxidation | 1.36E-02 |
